# Multimodal large language models address clinical queries in laryngeal cancer surgery: a comparative evaluation of image interpretation across different models

**DOI:** 10.1097/JS9.0000000000002234

**Published:** 2025-01-27

**Authors:** Bingyu Liang, Yifan Gao, Taibao Wang, Lei Zhang, Qin Wang

**Affiliations:** a Department of Otolaryngology Head and Neck Surgery, The First Affiliated Hospital of Anhui Medical University, Hefei, Anhui Province, China; b School of Biomedical Engineering, Division of Life Sciences and Medicine, University of Science and Technology of China, Hefei, Anhui Province, China; c Shanghai Innovation Institute, Shanghai, China (Q.Wang)

## Abstract

**Background and objectives:**

Recent advances in multimodal large language models (MLLMs) have shown promise in medical image interpretation, yet their utility in surgical contexts remains unexplored. This study evaluates six MLLMs’ performance in interpreting diverse imaging modalities for laryngeal cancer surgery.

**Methods:**

We analyzed 169 images (X-rays, CT scans, laryngoscopy, and pathology findings) from 50 patients using six state-of-the-art MLLMs. Model performance was assessed across 1084 clinically relevant questions by two independent physicians.

**Results:**

Claude 3.5 Sonnet achieves the highest accuracy (79.43%, 95% CI: 77.02%-81.84%). Performance varied significantly across imaging modalities and between commercial and open-source models, with a 19-percentage point gap between the best commercial and open-source solutions.

**Conclusion:**

Advanced MLLMs show promising potential as clinical decision support tools in laryngeal cancer surgery, while performance variations suggest the need for specialized model development and clinical workflow integration. Future research should focus on developing specialized MLLMs trained on large-scale multi-center laryngeal cancer datasets.

**HIGHLIGHTS**

- Six multimodal large language models (MLLMs) were evaluated across 6 image types, 169 images, and 1084 open-ended clinical questions in laryngeal cancer surgery.
- Advanced MLLMs demonstrate high accuracy (up to 79.43%) in interpreting diverse image modalities, with commercial models outperforming open-source alternatives.
- MLLMs show potential to enhance clinical decision-making across the surgical timeline of laryngeal cancer, from preoperative planning to post-operative care.

## Introduction

Recent advances in multimodal large language models (MLLMs) represent a paradigm shift in artificial intelligence, distinguished by their ability to engage in natural conversation about images. Unlike traditional systems that typically output predefined classifications or measurements, these general-purpose models can interpret images through interactive dialogue. MLLMs have demonstrated significant potential in medical image understanding, exhibiting impressive performance in tasks such as answering clinical questions^[1,2]^, disease classification^[3,4]^, and report interpretation^[5]^. Recent studies^[6]^ have also revealed their capacity to simulate stepwise clinical reasoning processes to some extent, indicating their applicability in diverse medical scenarios.

In surgical specialties, the ability to accurately interpret multiple image modalities is crucial for treatment planning and execution. Laryngeal cancer is one of the most prevalent types of head and neck cancers^[7]^, where precise interpretation of diverse image findings directly influences surgical approach and outcomes^[8]^. The comprehensive assessment of laryngeal cancer relies on complementary image modalities: laryngoscopy for primary lesion visualization, CT imaging for evaluating tumor extension and nodal status, radiography for detecting esophageal involvement, and pathological examination for definitive histological diagnosis.

While artificial intelligence has broadly demonstrated promise in enhancing diagnostic accuracy^[9,10]^, the potential utility of MLLMs in complex surgical contexts, specifically laryngeal cancer management, remains unexplored. This study aims to bridge this knowledge gap by evaluating the performance of six leading MLLMs in interpreting multiple image modalities and pathology findings crucial for laryngeal cancer surgical management. We hypothesize that MLLMs can interpret diverse image modalities and pathological data to provide accurate and clinically relevant insights for laryngeal cancer surgical management, potentially enhancing decision-making throughout the surgical process.

## Methods

**Figure 1.**
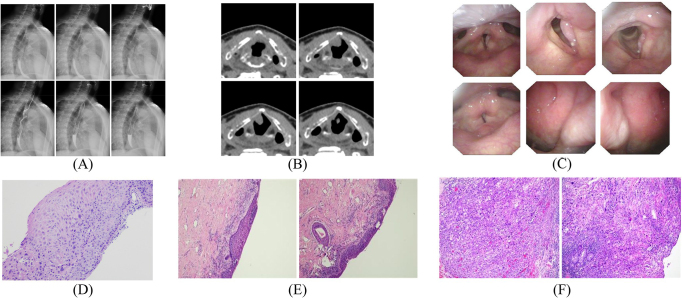
Examples of different image types used in laryngeal cancer assessment. (A) X-ray, (B) CT scan, (C) laryngoscopy image, (D) pre-operative, (E) intra-operative, and (F) post-operative pathology images.

To test our hypothesis, we collected a private dataset of multimodal images related to laryngeal cancer from 50 patients during routine clinical visits. The dataset comprised 169 images of various types, including X-rays, CT scans, laryngoscopy images, and pre-, intra-, and post-operative pathology images. Images with resolution below 128 × 128 pixels were excluded to ensure reliable model performance. It’s worth noting that not all patients had images for every modality. To facilitate analysis of sequential imaging data, such as CT and X-ray scans, we merged multiple slices into a single large image, preserving the sequential nature of the data. Examples of the images are illustrated in Figure 1. The image data was sourced from an ethically approved database (approval number 5101293), and this secondary analysis of fully anonymized data was granted exemption from additional ethical approval and informed consent requirements by the institutional ethics committee.

We assessed both commercial (GPT-4o, Claude 3.5 Sonnet, Gemini 1.5 Pro) and open-source (LLaVA-Med, InternVL, HuatuoGPT-Vision) models on their ability to accurately answer clinical queries based on the selected imaging and pathological data.

**Figure 2.**
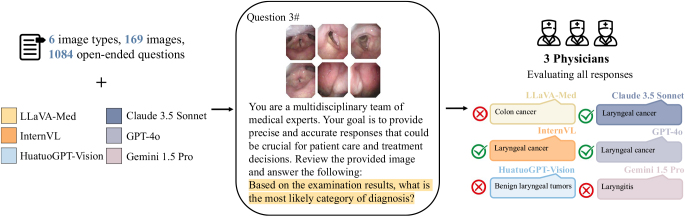
Illustration of textual and visual inputs to multimodal large language models (MLLMs). We input these questions along with their corresponding images into each of the six MLLMs and collected their responses.

The overall study design is illustrated in Figure 2. For each image type, we formulated a set of clinically relevant questions, totaling 1084 questions across all images, that reflected the key considerations in laryngeal cancer surgical planning and management. The complete list of questions is provided in Supplementary Table 1 (http://links.lww.com/JS9/D795).

The accuracy of responses was validated against clinical case reports, with two independent physicians reviewing for factual correctness and clinical appropriateness. In cases where there was a discrepancy between the two evaluators, a senior physician was consulted to make the final determination. To maintain consistency and minimize bias, the evaluation process was conducted blindly, with evaluators unaware of which MLLM generated each response. We calculated the percentage of correct responses and 95% confidence intervals (CI) for each model across all image types.

## Results

**Figure 3.**
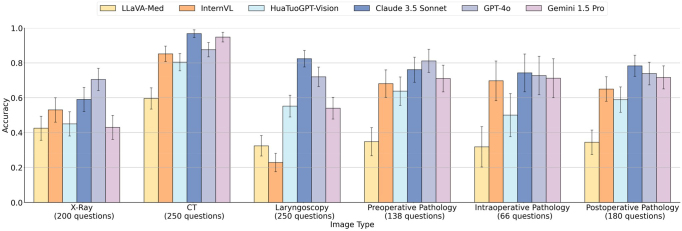
Performance of multimodal large language models (MLLMs) on different image types.

Figure 3 illustrates the performance of the six state-of-the-art MLLMs in interpreting diverse imaging and pathological data relevant to laryngeal cancer surgery. Claude 3.5 Sonnet exhibited the highest overall accuracy at 79.43% (95% CI, 77.02%–81.84%), closely followed by GPT-4o at 76.85% (95% CI, 74.33%–79.36%). Gemini 1.5 Pro showed moderate performance with an accuracy of 67.53% (95% CI, 64.74%–70.32%). Among the open-source models, HuatuoGPT-Vision and InternVL demonstrated similar capabilities, achieving accuracy rates of 60.52% (95% CI, 57.60%–63.43%) and 58.39% (95% CI, 55.46%–61.33%), respectively. LLaVA-Med had the lowest overall accuracy at 41.14% (95% CI, 38.21%–44.08%).

The performance gap between the best-performing commercial model (Claude 3.5 Sonnet) and the top open-source model (HuatuoGPT-Vision) was substantial, with a difference of approximately 19 percentage points. Subgroup analysis revealed differential performance across image modalities. Claude 3.5 Sonnet achieved an impressive 96.80% accuracy (95% CI, 94.60%–99.00%) on CT scans, while its performance on X-rays was 59.00% (95% CI, 52.12%–65.88%).

## Discussion

This study provides a comprehensive evaluation of the performance of six state-of-the-art MLLMs in interpreting diverse imaging and pathological data relevant to laryngeal cancer surgery. Our findings reveal significant variability in the capabilities of these models, with important implications for their potential integration into clinical practice.

The superior performance of commercial models, particularly Claude 3.5 Sonnet and GPT-4o, with overall accuracies exceeding 75%, demonstrates the potential of advanced MLLMs to support clinical decision-making in laryngeal cancer surgery. The observed variations in model performance across different image modalities provide valuable insights into the strengths and limitations of current MLLMs. For instance, while performance on CT scans was consistently high across models, there was greater variability in the interpretation of laryngoscopy images and X-rays. This suggests that further refinement may be needed to enhance MLLM performance on these specific modalities.

The analysis of pathology interpretations across pre-, intra-, and post-operative stages reveals interesting patterns. The relatively consistent performance of top models across these stages is encouraging, suggesting potential applicability throughout the surgical timeline. However, the slight variations in accuracy across these stages warrant further investigation to ensure reliable support at all points of care.

While the results are promising, we acknowledge their limitations. Importantly, these tools are designed to support, not replace, the clinical judgment of healthcare professionals. The value of clinical expertise remains paramount in medical decision-making. Our evaluation protocol utilized predefined questions that may not fully encapsulate the intricate nature of real-world clinical scenarios. Moreover, our single institution dataset may not fully represent the diverse patient demographics seen in clinical practice, and the model’s performance could vary across different patient populations and anatomical variations.

## Conclusion

We conducted a comprehensive evaluation of six state-of-the-art MLLMs in interpreting diverse imaging and pathological data relevant to laryngeal cancer surgery. Our study demonstrates that advanced MLLMs, particularly commercial models, show promising performance in accurately answering clinical queries related to laryngeal cancer management. These models could serve as rapid consultation tools during clinical decision-making, providing additional diagnostic perspectives in complex cases. Future research should focus on developing specialized MLLMs trained on large-scale multi-center laryngeal cancer image datasets and validating their integration into clinical workflows to enhance reliability and practical utility in patient care.

## Data Availability

The data are available upon reasonable request.
